# Disparities in Exposure to Tobacco on Television or Streaming Platforms

**DOI:** 10.1001/jamanetworkopen.2024.27781

**Published:** 2024-08-22

**Authors:** Henry K. Onyeaka, Onyema G. Chido-Amajuoyi, Itunu Sokale, Kobi V. Ajayi, A Eden Evins, Hermioni L. Amonoo, Sanjay Shete

**Affiliations:** 1 Department of Psychiatry, Harvard Medical School, Boston, Massachusetts; 2 Department of Psychiatry, Massachusetts General Hospital, Boston; 3 Department of Psychiatry, Mclean Hospital, Belmont, Massachusetts; 4 Fred Hutchinson Cancer Center, Seattle, Washington; 5 Department of Medicine, University of Washington, Seattle; 6 Department of Medicine, Baylor College of Medicine, Houston, Texas; 7 Department of Health Behavior, School of Public Health, Texas A & M University, College Station; 8 Department of Psychiatry, Brigham and Women’s Hospital, Boston, Massachusetts; 9 Department of Psychosocial Oncology and Palliative Care, Dana-Farber Cancer Institute, Boston, Massachusetts; 10 Department of Biostatistics, The University of Texas MD Anderson Cancer Center, Houston; 11 Division of Cancer Prevention and Population Sciences, The University of Texas MD Anderson Cancer Center, Houston

## Abstract

**Importance:**

With the rise in popularity of streaming platforms concerns about exposure to tobacco advertising and promotion have emerged. While tobacco marketing and promotion through traditional television (TV) media channels has been extensively studied, less is known about exposure to tobacco through TV or streaming platforms and its associated factors.

**Objective:**

To examine the prevalence and factors associated with exposure to tobacco products advertised, marketed, or promoted on TV or streaming platforms among US adults.

**Design, Setting, and Participants:**

This cross-sectional study used data from the National Cancer Institute’s Health Information National Trends Survey (HINTS 6), conducted from March 7 to November 8, 2022. The nationally representative survey included noninstitutionalized civilian US adults.

**Main Outcomes and Measures:**

The primary outcome was self-reported exposure to tobacco advertisements, marketing, or promotion on TV or streaming platforms in the past 3 months. Factors associated with exposure were explored using multivariable survey logistic regression.

**Results:**

The study included 5775 participants (3415 females [weighted percentage, 50.5%], 970 Hispanic individuals [weighted percentage, 16.9%], 872 non-Hispanic Black or African American individuals [11.1%], 3144 non-White individuals [61.5%], and 632 individuals who currently smoke [12.0%]). The estimated exposure to tobacco advertisements, marketing, or promotion on television or streaming platforms was 12.4% (95% CI, 10.8%-14.2%). Multivariable logistic regression analysis revealed that exposure odds were higher among those who had a level of education of high school or less (adjusted odds ratio [aOR], 1.60; 95% CI, 1.08-2.37), individuals who currently smoke (aOR, 1.85; 95% CI, 1.06-3.25), non-Hispanic Black or African American respondents (aOR, 2.20; 95% CI, 1.40-3.45) and Hispanic respondents (aOR, 1.58; 95% CI, 1.04-2.42).

**Conclusions and Relevance:**

In this study of the prevalence of exposure to tobacco advertisements on TV or streaming platforms among US adults, disparities in exposure by race or ethnicity, education level, and smoking status were identified. These findings underscore the need for targeted public health interventions and regulation to address these disparities and reduce the impact of tobacco advertisements on vulnerable populations.

## Key Points

**Question:**

What are the prevalence and sociodemographic factors associated with exposure to tobacco on television (TV) or streaming platforms among US adults?

**Findings:**

This cross-sectional study of 5775 US adults found that exposure to tobacco on TV or streaming platforms was estimated at 12.4%. The odds of exposure were higher among non-Hispanic Black or African American respondents, Hispanic respondents, those with lower educational attainment, and individuals who currently smoke.

**Meaning:**

These findings suggest that exposure to tobacco on TV or streaming platforms may differ by key sociodemographic and behavioral characteristics, indicating the need for targeted public health interventions and regulation to address disparities in exposure and ultimately reduce tobacco-related health disparities.

## Teaser

This cross-sectional study examines the prevalence and factors associated with exposure to tobacco products advertised, marketed, or promoted on television or streaming platforms among US adults.

## Introduction

Streaming platforms have undoubtedly revolutionized the entertainment industry, experiencing exponential growth in popularity. As of 2023, approximately 247 million individuals globally were paid subscribers to a single streaming platform alone.^1^ However, with this surge in popularity comes a growing concern for untoward public health exposures, specifically exposure to tobacco products.

Tobacco advertising, marketing, and promotion, such as through depictions of smoking in shows and movies, are strongly associated with tobacco use.^2,3^ Identifying this problem, Article 13 of World Health Organization (WHO) Framework Convention on Tobacco Control addresses measures aimed at preventing exposure to tobacco advertising and promotion.^4^ Under this article, WHO recommends a complete ban on all forms of tobacco advertising and promotion. In the US, a ban on cigarette advertisements on TV and radio, specifically those stations broadcasting on airwaves regulated by the Federal Communications Commission, was enacted in 1971.^5,6^ However, these regulations do not extend to the nuanced portrayals of tobacco in modern streaming content, allowing tobacco companies to subtly influence viewers, circumventing existing advertising restrictions.^7,8^ Moreover, it remains challenging to determine whether these portrayals are backed by tobacco companies or are independent artistic choices, leaving depictions in public media largely unregulated.

The association of tobacco advertising and promotion with tobacco use behaviors has been extensively studied with traditional TV media.^2,9^ However, the reach and impact of tobacco products being advertised, marketed, or promoted on streaming platforms remains an understudied area despite the exponential growth of this mode of content consumption. A report by the Truth Initiative analyzed popular shows on streaming platforms and found a high prevalence of exposure to smoking, despite a pledge by a streaming platform to eliminate smoking portrayals in new original programming aimed at younger viewers.^10^

Given the established association between tobacco advertising and increased tobacco use,^3^ documented disparities in exposure to tobacco advertising,^11^ and the scant research on exposure to tobacco on emerging channels, such as TV or streaming platforms, this study aims to investigate both the prevalence and sociodemographic factors associated with exposure to tobacco advertising and promotion on TV or streaming platforms within a nationally representative sample of US adults. The findings of this study could help inform targeted public health interventions to mitigate disparities in tobacco-related outcomes.

## Methods

### Study Population, Design, and Setting

We examined data from the National Cancer Institute’s Health Information National Trends Survey (HINTS 6), conducted from March 7 through November 8, 2022. HINTS 6 is a nationally representative survey of noninstitutionalized civilian US adults. The sample frame for HINTS 6 was derived using the Marketing Systems Group database of addresses and included all nonvacant residential addresses in the US. In this sampling frame, addresses were grouped into low–racial and ethnic minority and high–racial and ethnic minority strata. Similar to previous iterations of the HINTS 6,^12^ the high-minority strata were oversampled to enhance the accuracy of estimates for this population. An equal-probability method was used to select addresses within each stratum, and 1 adult per sampled household was selected to participate in the survey. The survey response rate of HINTS 6 was 28.1% and was comparable with previous cycles of HINTS.^12^ Written informed consent was obtained from study participants. HINTS 6 data are publicly available and was approved by the institutional review board at Westat and classified as exempt from review by the US National Institutes of Health Office of Human Subjects Research Protections because the data were deidentified. This cross-sectional study followed the Strengthening the Reporting of Observational Studies in Epidemiology (STROBE) reporting guideline.^13^ The HINTS 6 Methodology Report^14^ contains additional information about the sampling and weighting process.

### Outcome Measures

The outcome of interest was the self-reported exposure to tobacco advertisement, marketing, or promotion on TV or streaming platforms. Specifically, respondents were asked, “During the past 3 months, have you noticed or heard tobacco products being advertised, marketed, or promoted in any of the following places?” Respondents were required to mark all that apply, and the response of interest for this study was those endorsed having seen or heard tobacco products being marketed, advertised, or promoted on TV or streaming platforms. Among the survey respondents, more than 90% responded to the question on exposure to tobacco advertisements on TV or streaming platforms.

### Participant Characteristics

Several factors were considered in the evaluation of exposure to tobacco on TV or streaming platforms including age, sex, race and ethnicity, level of education, rural-urban residence, household annual income, and smoking status. Age was grouped into 4 categories as follows: 18 to 34 years (reference category), 35 to 49 years, 50 to 64 years, and 65 years or older. Race and ethnicity was self-reported and was categorized as Hispanic, non-Hispanic Black or African American, non-Hispanic White (reference category), and other (ie, non-Hispanic American Indian or Alaska Native, non-Hispanic Asian, non-Hispanic Native Hawaiian or Other Pacific Islander, and non-Hispanic multiple races). Level of education was grouped into 3 categories: high school graduate or less, post–high school or some college (a combination of post–high school training other than college and some college training), and college graduate or postgraduate (reference category). The residence was defined using the US Department of Agriculture’s 2003 Rural-Urban Continuum Codes. Codes 1 to 3 were designated as urban (reference category), while codes 4 to 9 were categorized as rural. Household annual income was categorized as less than $20 000 (reference category), $20 000 to $34 999, $35 000 to $49 999, $50 000 to $74 999, and $75 000 or more. Smoking status was ascertained from the question, “Have you smoked at least 100 cigarettes in your entire life?” Those who answered no were categorized as individuals who never smoked (reference category). Among those who answered yes, a follow-up question was asked: “Do you now smoke cigarettes every day, some days, or not at all?” Those who answered not at all were categorized as individuals who previously smoked, while others were considered individuals who currently smoke.

### Statistical Analysis

Prevalence of exposure to the tobacco products being advertised, marketed, or promoted on TV or streaming platforms was estimated for the overall sample, as well as by age, sex, race and ethnicity, level of education, rural-urban residence, household annual income, and tobacco use characteristics. Because individuals who never smoked are a vulnerable group who may initiate smoking through targeted tobacco product marketing,^15^ we examined exposure prevalence by sociodemographic characteristics among a subpopulation of individuals who never smoked.

Next, factors associated with exposure to the tobacco products being advertised, marketed, or promoted on TV or streaming platforms were explored using separate multivariable survey logistic regression. In the first model, the odds of exposure were calculated based on sociodemographic variables, with individuals who did not report seeing advertisements on TV or streaming platforms serving as the reference group. The second model estimated the odds of exposure based on sociodemographic variables, using individuals who did not report seeing any tobacco product advertisements at all as the reference group.

The statistical significance level was defined as 2-sided *P* < .05. All statistical analyses were performed using the svy command in the Stata version 17.0 (StataCorp). Final person weights and jack-knife replicate weights provided within the HINTS 6 dataset were used to derive national-level estimates. Analyses were conducted from October 2023 to February 2024.

## Results

The overall sample of 5775 respondents included 3415 females (weighted percentage, 50.5%), 970 Hispanic individuals (16.9%), 872 non-Hispanic Black or African American individuals (11.1%), 3144 non-Hispanic White individuals (61.5%), and 632 individuals who currently smoke (12.0%) (Table 1). The estimated exposure to tobacco advertisements, marketing, or promotion on TV or streaming platforms was 12.4% (95% CI, 10.8%-14.2%). Exposure was highest among those aged 35 to 49 years (13.4%; 95% CI, 10.8%-16.5%), those with a high school education or less (16.4%; 95% CI, 13.7%-19.6%), non-Hispanic Black or African American respondents (19.4%; 95% CI, 15.4%-24.2%), those with a household annual income less than $20 000 (17.6%; 95% CI, 14.1%-21.9%), and among individuals who currently smoke (17.0%; 95% CI, 11.4%-24.5%). The other response options for exposure to tobacco advertisements, marketing, or promotion and how often they were endorsed are reported in the eTable in Supplement 1.

**Table 1.** Exposure to Tobacco Advertisements, Marketing, or Promotion on Television or Streaming Platforms by Sociodemographic Characteristics and Smoking Status^a^

Sample characteristics	Total, No. (weighted %)	Exposure to tobacco on television or streaming platforms, weighted % (95% CI) [No.]	
		No^b^	Yes^c^
Participants, No.	5775	5048	727
Sex			
Female	3415 (50.5)	86.7 (84.5-88.6) [2984]	13.3 (11.4-15.5) [431]
Male	2271 (49.5)	88.8 (85.8-91.2) [1991]	11.2 (8.8-14.2) [280]
Age, y			
18-34	825 (25.3)	87.2 (81.3-91.5) [721]	12.8 (8.5-18.7) [104]
35-49	1132 (24.9)	86.6 (83.5-89.2) [991]	13.4 (10.8-16.5) [141]
50-64	1667 (27.8)	89.0 (86.7-91.0) [1453]	11.0 (9.0-13.3) [214]
≥65	2082 (22.0)	87.7 (85.7-89.4) [1827]	12.3 (10.6-14.3) [255]
Education			
High school or less	1385 (28.2)	83.6 (80.4-86.3) [1148]	16.4 (13.7-19.6) [237]
Some college	1637 (39.1)	87.8 (84.5-90.5) [1414]	12.2 (9.5-15.5) [223]
College graduate or more	2663 (32.7)	90.6 (88.5-92.3) [2411]	9.4 (7.7-11.5) [252]
Household annual income, $			
<20 000	916 (14.2)	82.4 (78.1-85.9) [755]	17.6 (14.1-21.9) [161]
20 000-34 999	713 (11.1)	83.0 (78.0-87.0) [597]	17.0 (13.0-22.0) [116]
35 000-49 999	713 (11.8)	89.5 (85.9-92.2) [616]	10.5 (7.8-14.1) [97]
50 000-74 999	918 (18.2)	90.5 (87.5-92.9) [816]	9.5 (7.1-12.5) [102]
≥75 000	2132 (44.6)	88.6 (85.3-91.3) [1927]	11.4 (8.7-14.7) [205]
Race and ethnicity^d^			
Hispanic	970 (16.9)	83.6 (79.2-87.1) [812]	16.4 (12.9-20.8) [158]
Non-Hispanic Black or African American	872 (11.1)	80.6 (75.8-84.6) [699]	19.4 (15.4-24.2) [173]
Non-Hispanic White	3144 (61.5)	90.3 (87.6-92.5) [2862]	9.7 (7.5-12.4) [282]
Other^e^	459 (10.5)	88.3 (80.0-93.4) [409]	11.7 (6.6-19.9) [50]
Residence			
Urban	5023 (87.6)	87.6 (85.8-89.3) [4393]	12.4 (10.7-14.2) [630]
Rural	752 (12.4)	87.4 (82.9-90.8) [655]	12.6 (9.2-17.1) [97]
Smoking status			
Never	3662 (65.6)	88.7 (87.0-90.1) [3196]	11.3 (9.9-13.0) [466]
Former	1430 (22.4)	87.3 (83.2-90.5) [1272]	12.7 (9.5-16.8) [158]
Current	632 (12.0)	83.0 (75.5-88.6) [535]	17.0 (11.4-24.5) [97]

^a^ Participants were asked to assess their exposure to tobacco advertisements on television or streaming platforms with the following prompt: “During the past 3 months, have you noticed or heard tobacco products being advertised, marketed, or promoted in any of the following places? Mark all that apply.” The response of interest was “On television or streaming platforms (including Netflix or Hulu).”

^b^ No indicates all those who reported no exposure to tobacco advertisements on television or streaming platforms during the past 3 months and all those who did not report exposure to tobacco products at all in the past 3 months.

^c^ Yes indicates all those who reported exposure to tobacco advertisements on television or streaming platforms during the past 3 months.

^d^ Race was assessed as part of the sociodemographic characteristics of study participants and was self-reported.

^e^ Others include non-Hispanic American Indian or Alaska Native, non-Hispanic Asian, non-Hispanic Native Hawaiian or Other Pacific Islander, and non-Hispanic multiple races.

Among the subpopulation of individuals who never smoked, we found that 11.3% (95% CI, 9.9%-13.0%) reported being exposed to tobacco products being advertised, marketed, or promoted on TV or streaming platforms (Table 2). Corresponding with findings in the general population, exposure proportion among individuals who never smoked was highest among those with a high school education or less (15.0%; 95% CI, 11.8%-18.9%), non-Hispanic Black or African American respondents (17.5%; 95% CI, 13.1%-23.1%), and those with a household annual income between $20 000 to $35 000 (16.5%; 95% CI, 11.3%-23.5%). However, unlike the findings in the general population, exposure among individuals who never smoked were highest among respondents aged 65 years or more (13.2%; 95% CI, 10.5%-16.5%).

**Table 2.** Exposure to Tobacco Advertisements, Marketing, or Promotion on Television or Streaming Platforms by Sociodemographic Characteristics Among Individuals Who Never Smoked^a^

Sample characteristics	Total, No. (weighted %)	Exposure to tobacco on television or streaming platforms, weighted % (95% CI) [No.]	
		No^b^	Yes^c^
Participants, No.	3662	3196	466
Sex			
Female	2255 (51.5)	86.8 (84.4-88.9) [1967]	13.2 (11.1-15.6) [288]
Male	1348 (48.5)	91.1 (88.6-93.1) [1184]	8.9 (6.9-11.4) [164]
Age, y			
18-34	687 (31.4)	90.2 (86.8-92.8) [603]	9.8 (7.2-13.4) [84]
35-49	760 (24.8)	89.8 (85.9-92.7) [675]	10.2 (7.3-14.1) [85]
50-64	1026 (25.0)	87.5 (84.5-90.0) [882]	12.5 (10.0-15.5) [144]
≥65	1140 (18.8)	86.8 (83.5-89.4) [998]	13.2 (10.5-16.5) [142]
Education			
High school or less	757 (26.0)	85.0 (81.1-88.2) [621]	15.0 (11.8-18.9) [136]
Some college	922 (35.8)	89.0 (85.5-91.7) [790]	11.0 (8.3-14.5) [132]
College graduate or more	1921 (38.2)	90.9 (88.8-92.6) [1736]	9.1 (7.4-11.2) [185]
Household annual income, $			
<20 000	496 (12.7)	86.0 (81.8-89.4) [414]	14.0 (10.6-18.2) [82]
20 000-34 999	397 (10.2)	83.5 (76.5-88.7) [327]	16.5 (11.3-23.5) [70]
35 000-49 999	438 (11.9)	89.4 (84.8-92.7) [380]	10.6 (7.3-15.2) [58]
50 000-74 999	578 (18.4)	90.6 (87.1-93.2) [505]	9.4 (6.8-12.9) [73]
≥75 000	1506 (46.8)	89.9 (87.0-92.2) [1356]	10.1 (7.8-13.0) [150]
Race and ethnicity^d^			
Hispanic	729 (20.0)	84.7 (79.8-88.6) [616]	15.3 (11.4-20.2) [113]
Non-Hispanic Black or African American	574 (11.3)	82.5 (76.9-86.9) [460]	17.5 (13.1-23.1) [114]
Non-Hispanic White	1836 (57.4)	91.4 (88.9-93.4) [1667]	8.6 (6.6-11.1) [169]
Other^e^	335 (11.3)	90.8 (84.5-94.7) [303]	9.2 (5.3-15.5) [32]
Residence			
Urban	3219 (89.0)	88.9 (87.2-90.5) [2816]	11.1 (9.5-12.8) [403]
Rural	443 (11.0)	86.2 (82.2-89.4) [380]	13.8 (10.6-17.8) [63]

^a^ Participants were asked to assess their exposure to tobacco advertisements on television or streaming platforms with the following prompt: “During the past 3 months, have you noticed or heard tobacco products being advertised, marketed, or promoted in any of the following places? Mark all that apply.” The response of interest was “On television or streaming platforms (including Netflix or Hulu).”

^b^ No indicates all those who reported no exposure to tobacco advertisements on television or streaming platforms during the past 3 months and all those who did not report exposure.

^c^ Yes indicates all those who reported exposure to tobacco advertisements on television or streaming platforms during the past 3 months.

^d^ Race was assessed as part of the sociodemographic characteristics of study participants and was self-reported.

^e^ Others include non-Hispanic American Indian or Alaska Native, non-Hispanic Asian, non-Hispanic Native Hawaiian or Other Pacific Islander, and non-Hispanic multiple races.

In the general population, with individuals who did not report seeing advertisements on TV or streaming platforms serving as the reference group, our multivariable logistic regression analysis found that, following adjustment, exposure odds were higher among those who had a level of education of high school or less (adjusted odds ratio [aOR], 1.60; 95% CI, 1.08-2.37) compared with those who had a college or postgraduate degree (Table 3). The odds of exposure on TV or streaming platforms were higher among individuals who currently smoke than individuals who do not smoke (aOR, 1.85; 95% CI, 1.06-3.25). Exposure odds were also higher among non-Hispanic Black or African American respondents (aOR, 2.20; 95% CI, 1.40-3.45) and Hispanic respondents (aOR, 1.58; 95% CI, 1.04-2.42) compared with non-Hispanic White respondents. Similar findings were observed when using individuals who did not report seeing any tobacco product advertisements at all as the reference group (Table 4).

**Table 3.** Multivariable Regression Analysis of the Association Between Sociodemographic Characteristics and Exposure to Tobacco Advertisements, Marketing, or Promotion on Television or Streaming Platforms Among US Adults^a^

Sample characteristics	Adjusted OR (95% CI)^a^	*P* value
Sex		
Female	1 [Reference]	NA
Male	0.85 (0.60-1.20)	.35
Age, y		
18-34	1 [Reference]	NA
35-49	0.91 (0.48-1.73)	.78
50-64	0.74 (0.42-1.30)	.28
≥65	0.85 (0.44-1.63)	.62
Education		
High school or less	1.60 (1.08-2.37)	.02
Some college	1.16 (0.79-1.69)	.45
College graduate or more	1 [Reference]	NA
Household annual income, $		
<20 000	1 [Reference]	NA
20 000-34 999	0.99 (0.63-1.58)	.99
35 000-49 999	0.71 (0.43-1.17)	.18
50 000-74 999	0.64 (0.40-1.01)	.06
≥75 000	1.00 (0.63-1.60)	.97
Race and ethnicity		
Hispanic	1.58 (1.04-2.42)	.04
Non-Hispanic Black or African American	2.20 (1.40-3.45)	.001
Non-Hispanic White	1 [Reference]	NA
Other^b^	1.25 (0.60-2.61)	.54
Residence		
Urban	1 [Reference]	NA
Rural	0.95 (0.55-1.62)	.84
Smoking status		
Never	1 [Reference]	NA
Former	1.34 (0.85-2.13)	.20
Current	1.85 (1.06-3.25)	.03

Abbreviations: NA, not applicable; OR, odds ratio.

^a^ Reference group included participants who did not report seeing tobacco product advertisement, marketing or promotion on TV or streaming platforms, which included those who did not see any tobacco product adverts at all and those who reported seeing tobacco product adverts on other platforms but not TV or streaming platforms (n = 5775).

^b^ Others include non-Hispanic American Indian or Alaska Native, non-Hispanic Asian, non-Hispanic Native Hawaiian or Other Pacific Islander, and non-Hispanic multiple races.

**Table 4.** Multivariable Regression Analysis of the Association Between Sociodemographic Characteristics and Exposure to Tobacco Advertisements, Marketing, or Promotion on Television or Streaming Platforms Among US Adults^a^

Sample characteristics	Adjusted OR (95% CI)	*P* value
Sex		
Female	1 [Reference]	NA
Male	0.79 (0.58-1.13)	.20
Age, y		
18-34	1 [Reference]	NA
35-49	0.75 (0.40-1.40)	.35
50-64	0.61 (0.34-1.08)	.09
≥65	0.59 (0.29-1.12)	.10
Education		
High school or less	1.57 (1.08-2.29)	.02
Some college	1.11 (0.76-1.63)	.58
College graduate or more	1 [Reference]	NA
Household annual income, $		
<20 000	1 [Reference]	NA
20 000-34 999	1.25 (0.74-2.10)	.39
35 000-49 999	0.79 (0.47-1.34)	.38
50 000-74 999	0.62 (0.38-1.02)	.06
≥75 000	0.99 (0.64-1.54)	.99
Race and ethnicity		
Non-Hispanic White	1 [Reference]	NA
Hispanic	1.65 (1.02-2.70)	.05
Non-Hispanic Black or African American	2.25 (1.40-3.60)	.001
Other^b^	1.19 (0.55-2.54)	.65
Residence		
Urban	1 [Reference]	NA
Rural	1.06 (0.62-1.83)	.82
Smoking status		
Never	1 [Reference]	NA
Former	1.62 (1.00-2.63)	.05
Current	1.85 (1.06-3.25)	.03

Abbreviations: NA, not applicable; OR, odds ratio.

^a^ Reference group included participants who did not report seeing any tobacco product advertisements, marketing, or promotion at all in the past 3 months (n = 3847).

^b^ Others include non-Hispanic American Indian or Alaska Native, non-Hispanic Asian, non-Hispanic Native Hawaiian or Other Pacific Islander, and non-Hispanic multiple races.

## Discussion

In this nationally representative cross-sectional study of US adults, more than one-tenth of the population reported being exposed to tobacco advertisement, marketing, or promotion on TV or streaming platforms. More so, we demonstrated disparities in the odds of tobacco exposure on TV or streaming platforms by race or ethnicity, level of education, and smoking status.

We found that non-Hispanic Black or African American respondents and Hispanic respondents exhibited significantly higher odds of exposure to tobacco products being advertised, marketed, or promoted compared with White respondents. With the advent of smart TVs and streaming platforms, advertising has become much more streamlined with greater precision. Hence, tobacco promotion could be targeted at specific racial groups through programs and may worsen existing disparities in tobacco use. Given that type of TV and media consumption falls along racial or ethnic categories,^16,17,18^ these findings emphasize the need for a nuanced understanding of how tobacco-related content is consumed within different racial and ethnic groups. Addressing these disparities is critical for developing culturally sensitive public health campaigns that effectively reach and resonate with diverse communities, ultimately contributing to the reduction of tobacco-related health disparities.

Our results also indicated higher odds of exposure to tobacco-related content among individuals who currently smoke than individuals who do not smoke. This result aligned with existing literature, which suggests that tobacco companies often direct their advertising efforts toward individuals who are already engaged in smoking to reinforce and sustain their habit. For instance, 1 study highlighted that tobacco advertising is strategically designed to appeal to individuals who currently smoke, using cues that trigger cravings and reinforce smoking behavior.^19^ Additionally, the higher exposure among individuals who currently smoke may also be attributed to algorithms used by streaming platforms, which tailor content based on viewers’ past behaviors and preferences. Hence, streaming services can be leveraged for more tobacco cessation education programming and interventions among individuals who currently smoke and who frequently patronize streaming services.

Socioeconomic disparities exist in smoking prevalence.^20^ Prior studies have demonstrated a preponderance for tobacco promotion and advertising exposure among young adults using traditional media,^2,21,22^ but we found no association by age. On the contrary, our study corresponds with other studies that demonstrate disparities in tobacco advertisement exposure by level of education.^11^ These findings underscore the importance of tailoring public health interventions for tobacco cessation on streaming services by education status.

### Limitations

This study has limitations. Study findings are limited by the low-response bias associated with HINTS 6 and other population-based surveys. Additionally, we could not delineate the exposed population based on the type of streaming platform to which they were exposed. Specifically, study data did not include adolescents, a highly vulnerable risk population for tobacco advertising and promotion. Furthermore, the lack of data on the duration of exposure to TV and streaming services precludes our ability to assess whether the observed differences are due to targeted advertisements or varying viewing times across racial groups. Future studies should consider including these variables to provide a more comprehensive analysis.

Finally, the survey question is inherently subjective and conflates promotion, advertising, and marketing into a single category. Thus, the outcome measure depends on individual perceptions of what constitutes tobacco promotion, advertisements, or marketing, and some individuals may not interpret depictions of tobacco products in shows as falling within these categories. Given that streaming platforms use limited advertisements and prohibit tobacco product advertisements, most reported exposures are likely due to tobacco use depictions in shows and movies. This type of promotion is difficult to regulate, as it is unclear whether tobacco companies fund such depictions or are artistic choices by content developers. Consequently, the survey may not accurately capture explicit tobacco advertising.

## Conclusions

This cross-sectional study contributes to a growing body of evidence that suggests the potential impact of the evolving landscape of digital media on tobacco control by uncovering patterns of exposure to smoking advertisements, marketing, or promotion on TV or streaming platforms. Study findings call for regulation in the TV and streaming space, given its potential for exploitation. As these platforms continue to dominate the entertainment landscape, it becomes increasingly important to regulate content that may contribute to detrimental health outcomes associated with smoking. These insights can also inform public health interventions that aim to mitigate the impact of tobacco advertisements, marketing, or promotion on TV or streaming platforms, especially among vulnerable populations. Thus, these interventions ultimately contribute to broader efforts to promote a healthier and more equitable society.

## Notes

Supplement 1. **eTable. **Exposure to Tobacco Products Being Advertised, Marketed, or Promoted in Any of the Following Places During the Past 3 Months

Supplement 2.
Data Sharing Statement
