# Supplementary material for: Disparities in Exposure to Tobacco on Television or Streaming Platforms
Source: JAMA Netw Open. 2024 Aug 22;7(8):e2427781. doi: 10.1001/jamanetworkopen.2024.27781 (PMC11342133; doi:10.1001/jamanetworkopen.2024.27781)
Supplement: Supplement 1. — eTable. Exposure to Tobacco Products Being Advertised, Marketed, or Promoted in Any of the Following Places During the Past 3 Months [file jamanetwopen-e2427781-s001.pdf]

Supplemental Online Content

Onyeaka HK, Chido-Amajuoyi OG, Sokale I, et al. Disparities in exposure to tobacco on television or streaming platforms. *JAMA Netw Open*. 2024;7(8):e2427781. doi:10.1001/jamanetworkopen.2024.27781

eTable. Exposure to Tobacco Products Being Advertised, Marketed, or Promoted in Any of the Following Places During the Past 3 Months

This supplemental material has been provided by the authors to give readers additional information about their work.

© 2024 Onyeaka HK et al. *JAMA Network Open.*

eTable. Exposure to tobacco products being advertised, marketed, or promoted in any of the following places during the past 3 months

Exposure to tobacco products being **advertised, marketed, or promoted** in any of the following places during the past 3 months (Total, N = 5775)	Weighted %, 95% CI,
inside or outside stores that sell tobacco products (Including product displays and signs)?	35.5 (33.5, 37.5)
on billboards (including by the roadside, places like bus stops, or on trains)?	14.0 (12.6, 15.4)
at a pharmacy?	2.7 (2.2, 3.4)
in bars or restaurants?	6.4 (5.5, 7.5)
at events (including fairs, markets, festivals, sporting events or music concerts)?	6.1 (4.9, 7.5)
at temporary or mobile sales locations or kiosks (including shopping centers, parked in the street, other places, but not at specific events)?	5.2 (4.4, 6.2)
on radio?	6.7 (5.6, 8.0)
on social media (including Facebook, Twitter, TikTok, YouTube, or Instagram)?	7.6 (6.3, 9.1)
on other websites or online sources	4.8 (4.0, 5.7)
in print newspapers or magazines	8.1 (6.9, 9.4)
in the mail or an email sent to you?	4.3 (3.4, 5.5)
any other place	0.9 (0.3, 3.3)
Did not see any tobacco products being advertised, marketed or promoted at all	52.2 (59.7, 54.7)

© 2024 Onyeaka HK et al. *JAMA Network Open.*
